# Fabrication of crystals from single metal atoms

**DOI:** 10.1038/ncomms4851

**Published:** 2014-05-27

**Authors:** Nicolas P. E. Barry, Anaïs Pitto-Barry, Ana M. Sanchez, Andrew P. Dove, Richard J. Procter, Joan J. Soldevila-Barreda, Nigel Kirby, Ian Hands-Portman, Corinne J. Smith, Rachel K. O’Reilly, Richard Beanland, Peter J. Sadler

**Affiliations:** 1 Department of Chemistry, University of Warwick, Gibbet Hill Road, Coventry CV4 7AL, UK; 2 Department of Physics, University of Warwick, Gibbet Hill Road, Coventry CV4 7AL, UK; 3 Australian Synchrotron, 800 Blackburn Road, Clayton, Victoria 3168, Australia; 4 School of Life Sciences, University of Warwick, Gibbet Hill Road, Coventry CV4 7AL, UK

## Abstract

Metal nanocrystals offer new concepts for the design of nanodevices with a range of potential applications. Currently the formation of metal nanocrystals cannot be controlled at the level of individual atoms. Here we describe a new general method for the fabrication of multi-heteroatom-doped graphitic matrices decorated with very small, ångström-sized, three-dimensional (3D)-metal crystals of defined size. We irradiate boron-rich precious-metal-encapsulated self-spreading polymer micelles with electrons and produce, in real time, a doped graphitic support on which individual osmium atoms hop and migrate to form 3D-nanocrystals, as small as 15 Å in diameter, within 1 h. Crystal growth can be observed, quantified and controlled in real time. We also synthesize the first examples of mixed ruthenium–osmium 3D-nanocrystals. This technology not only allows the production of ångström-sized homo- and hetero-crystals, but also provides new experimental insight into the dynamics of nanocrystals and pathways for their assembly from single atoms.

## Abstract (web summary)

Metal nanocrystals are used in an ever growing list of applications, but precise control and understanding of their formation can be difficult. Here, the authors show a route that allows the controlled formation of metal nanocrystals to be carried out and observed at an atom-by-atom level.

## Graphical abstract

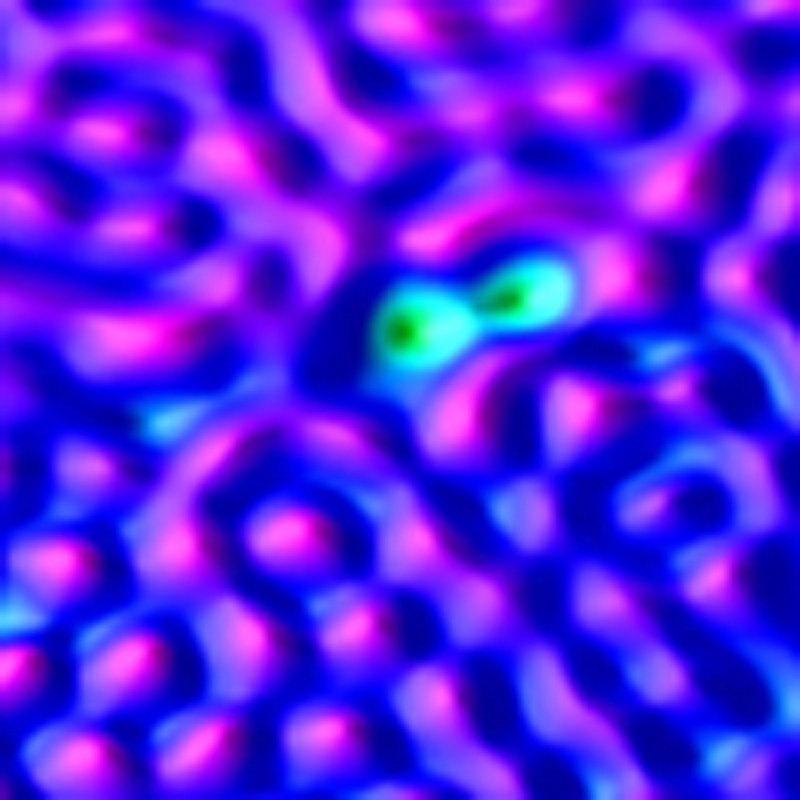

Tailoring nanoscopic objects is of importance for the production of the materials of the future, for example in medicine, industrial manufacturing, construction and space exploration^1^,^2^,^3^,^4^. The potential for use of metal nanocrystals has long been recognized. For example, colloidal gold was first made by Andreas Cassius in 1685 (ref. 5), and notably also by Michael Faraday in 1857 (ref. 6). Since then, a range of techniques has allowed the fabrication of nanocrystals, and these have allowed detailed investigation of their size-dependent properties^7^. The recent emergence of powerful methods such as high-resolution transmission electron microscopy, has enabled the design and the observation of a variety of nanometer-sized objects, from graphene matrices, to nanoparticles and nanotubes^8^,^9^.

The process of crystallization, the formation of microscopic periodic arrangements of atoms, ions and molecules, has long been a topic of intense fascination. It is 100 years since William Henry Bragg and his son William Lawrence Bragg revolutionized the characterization of crystalline solids by showing that the atomic structure of a crystal is related to its X-ray diffraction pattern^10^. Understanding the dynamics of crystal formation is also of great interest as the mode of assembly can influence the type of periodic lattice which is formed, and hence the properties of the resulting crystals. However, the direct observation of resolved single atoms, atom-by-atom cluster formation and the crystallization process for nanoscopic crystals is still an experimental frontier^11^,^12^,^13^,^14^,^15^. Currently, formation of such crystals cannot be controlled at the level of individual atoms^16^,^17^,^18^,^19^,^20^, and understanding the metal dynamics of nanocrystal formation relies mostly on computation^18^,^19^,^20^.

Here we introduce a new general method for the fabrication of multi-heteroatom-doped graphitic matrices decorated with very small, angstrom-sized, three-dimensional (3D)-metal crystals of defined size. This technology allows experimental observation of crystal assembly from single metal atoms and captures the dynamics of metal cluster formation in real space with atomic precision. We irradiate boron-rich precious metal-encapsulated self-spreading polymer micelles with electrons and produce, in real time, a doped graphitic support on which individual metal atoms hop and migrate to form 3D-nanocrystals, as small as 15 Å in diameter and within 1 h. Crystal growth can be observed, quantified and controlled in real time. Our procedure is exemplified by osmium, the densest of all metals^21^, and gives rise to osmium crystals, which are among the smallest reported^22^, embedded into a B/S-doped graphitic support. As an example of the generality of the procedure, we also synthesize the first examples of mixed ruthenium–osmium 3D-nanocrystals. This technology not only allows the production of angstrom-sized homo- and hetero-crystals, highly novel materials with potentially useful and unusual properties, but also provides new experimental insight into the dynamics of nanocrystals and pathways for their assembly from single atoms.

## Results

### Preparation of precursors

**Figure 1:**
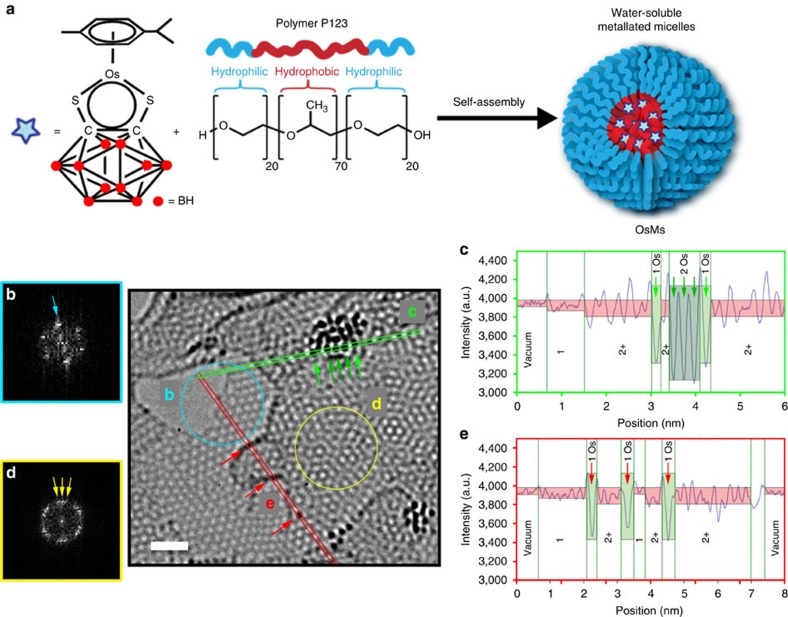
Micelle formation and analysis after irradiation with electrons. (**a**) Self-assembly of block copolymer micelles **OsMs** containing encapsulated osmium carborane complex (**b**–**e**): analysis of a typical area after degradation of the micelles by irradiation showing formation of a self-supporting graphitic matrix and individual osmium atoms (scale bar, 1 nm), characterized as follows: (**b**) Fast Fourier transform corresponding to single-sheet graphene-like material at the edges of the material, blue circle. (**c**) Intensity plot along the green line; the difference in thickness of the graphitic matrix (single layer versus 2 or more layers) is evident from the range of intensities (pink bands), similarly the difference between single and stacked Os atoms is evident for the cluster of atoms (green arrows), demonstrating its 3D nature. (**d**) Fast Fourier transform corresponding to a three-layer graphene-like material, yellow circle. (**e**) Detection of single Os atoms along the red line.

Our procedure involved the encapsulation of the organometallic half-sandwich Os^II^ arene complex [Os(*p*-cymene)(1,2-dicarba-*closo*-dodecarborane-1,2-dithiolate)] (**1**), a 16-electron complex^23^, which is highly hydrophobic^24^; in the water-soluble amphiphilic triblock copolymer P123 (ref. 25) at ambient temperature for 4 h to form **OsMs** micelles (Fig. 1a). The **OsMs** micelles were fully characterized by a range of techniques, including synchrotron small-angle X-ray scattering (SAXS) experiments (see Methods, Supplementary Figs 1–6, and Supplementary Table 1). These analyses demonstrated that complex **1** and polymer P123 self-assemble in solution to give core/shell micelles with very low-dispersity parameters (0.161), containing 52±6 P123 unimers, along with 52±11 complex **1**. They have a core diameter of 9.06±0.12 nm, and a shell diameter of 6.50±0.15 nm, giving a total-diameter of 15.56±0.27 nm. The data are summarized in Supplementary Table 1. **OsMs** micelles are dispersible in water. They contain a defined number of metal complexes, and are deformable on surfaces since P123 polymer forms stable Langmuir films at ambient temperature^26^. Thus, we deposited aqueous droplets ([**OsMs**]=1 mg ml^−1^) onto lacey carbon-coated grids to produce an unsupported film over the grid holes for examination by aberration-corrected high-resolution transmission electron microscopy (HR-TEM).

### Formation of a graphitic matrix and Os nanocrystals

We observed structural changes within the Pluronic film upon irradiation with the high-energy electron beam (80 keV; 1.9 pA cm^−2^ or 7.6 × 10^7^ electrons·nm^−2^ s^−1^). The emergence of atomic ordering within the self-supporting matrix consistent with a turbostratic graphitic structure was apparent within a few minutes, and a highly structured few-layer graphene lattice was evident after 50 min. An analysis of this matrix is shown in Fig. 1. The existence of individual layers of single-sheet graphene-like material at the edges of the material was demonstrated by fast Fourier transform (FFT) analysis (Fig. 1b), while few-layer graphene-like material was also visible (Fig. 1c shows a region with three layers). Along with these structural modifications of the self-supporting polymeric film, a rapid decomposition of the carborane-containing complex **1** was also observed (in less than 1 min), and Os atoms could be imaged either singly or as small-ordered clusters (Fig 1c,e), which is currently at the forefront of such experiments^9^,^17^,^27^,^28^.

**Figure 2:**
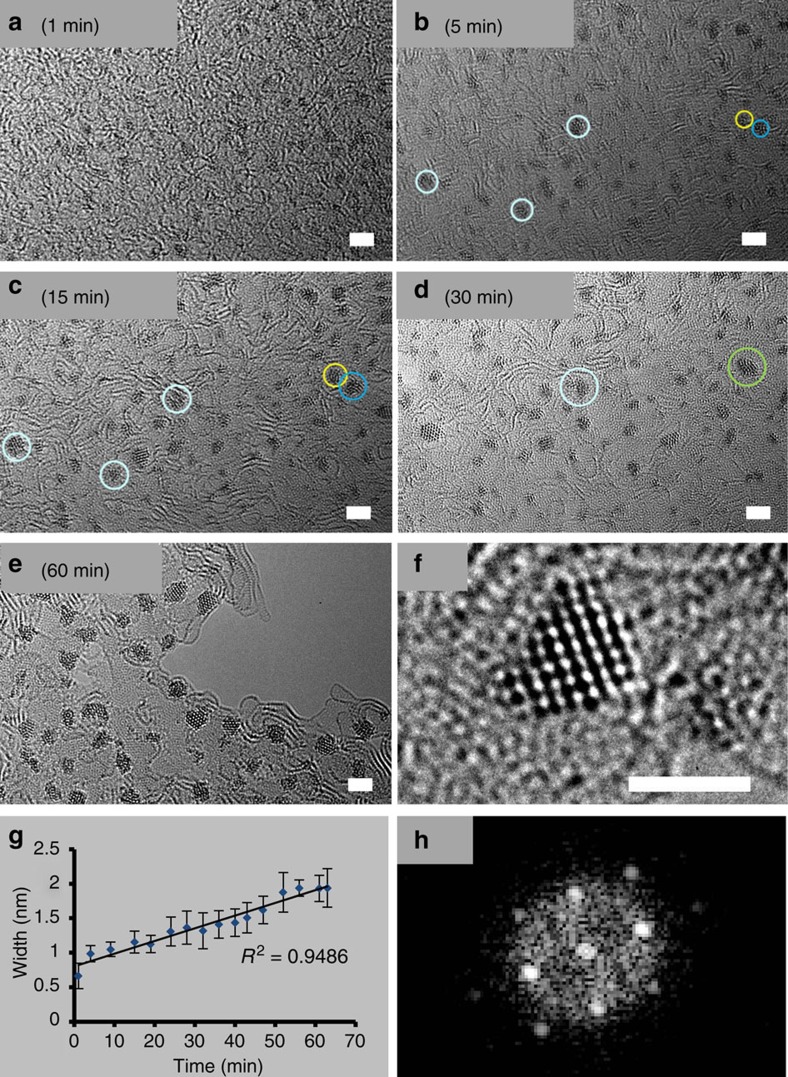
Time-dependent formation of Os nanocrystals on the graphitic matrix. (**a**–**d**) Migration of small clusters and their coalescence (for example, clusters in yellow and dark blue circles merge to give crystal in green circle) over a period 1–30 min; scale bars, 2 nm. (**e**) The abundance of nanocrystals after 60 min. (**f**) Typical example of an Os crystal of ca. 1.5 nm, formed after 30 min of irradiation, scale bar, 1.5 nm. (**g**) Width of the clusters/crystals versus time. The error bars are the standard deviations from the mean for measurements of the width of at least 20 clusters at each timepoint. (**h**) Fast Fourier transform analysis of the nanocrystal shown in **f**.

Electron energy loss spectroscopy not only confirmed the identity of the Os atoms, but also suggested that boron and sulfur from the carborane ligand in **1** are present in the graphitic matrix (Supplementary Fig. 7). Notably, sites with Os clusters (Supplementary Fig. 7c) appear to be close to both high-boron (Supplementary Fig. 7b,e,f) and high-sulfur sites (Supplementary Fig. 7a,d,f). Interestingly, boron and sulfur atoms are also seen throughout the entire matrix suggesting the formation of a multi-heteroatom-doped graphitic matrix. After 1 min of irradiation, clusters of *ca.* 0.7 nm were already visible (Fig. 2a). After 5 min of irradiation, dark areas containing *ca.* 15 atoms, became more organized and larger (*ca*. 1.0 nm, Fig. 2b). Further growth of the nanoclusters on the self-supporting matrix was evident after 15 min of irradiation (Fig. 2c), and after 30 min, osmium metal clusters with diameters of *ca.* 1.2–1.6 nm were visible (Fig. 2d). Growth was observed until *ca.* 60 min (Fig. 2), and the nanoclusters were seen to roll on the support in a series of images recorded within a minute (Supplementary Video 1). The movement of energized nanoclusters themselves results in contact with neighbouring particles, and eventual merging. Such cluster merging is illustrated in Supplementary Fig. 8 (also Supplementary Video 2). Interestingly, Zoberbier *et al.*^9^ reported that clusters of 20–60 Os atoms inside carbon nanotubes continuously change their shape under the influence of an 80 keV electron beam, and bind strongly to the inner surface, weakening C–C bonds and promoting gradual removal of carbon atoms.

Measurements of the length of the clusters indicate a linear relationship with time (Fig. 2g). This description is however only indicative owing to the 3D nature of the nanocrystals (Supplementary Video 3). Significantly, this *in situ* generation of Os atoms on a self-supporting graphitic matrix produces 3D nanoclusters that are crystalline^29^. This is illustrated by the fast Fourier transform (FFT) analysis (Fig. 2h) of the *ca.* 1.5 nm-diameter Os nanocrystal depicted in Fig. 2f. Supplementary Fig. 9 shows a montage of 10 osmium crystals along with their corresponding FFT analyses. In general, the Os crystals do not seem to have a simple hexagonal structure. Supplementary Fig. 10 shows two particles from the montage in Supplementary Fig. 9 with the closest appearance to the hexagonal structure and compares them with simulations. The c-axis view is distinctive, and the a-axis view is a good fit, with the correct interatomic distances. Nevertheless, we believe that most of the images do not match either of these two views, which are not the only ones with views that look relatively simple. The other views have more complex behaviour dependent on particle size and microscope focus. However, the average Os–Os distance measured over 85 different nanocrystals was determined to be 0.257±0.019 nm, close to that in crystals of bulk osmium metal (0.27048, nm as the nearest neighbor distance at 293.15 K)^30^,^31^. No change in Os–Os distance was observed during nanocrystal growth, as shown by the narrow standard deviation for the average Os–Os distance calculated from these 85 nanocrystals of various sizes (width between 1.5 and 2 nm).

### Mixed Os/Ru nanocrystals

**Figure 3:**
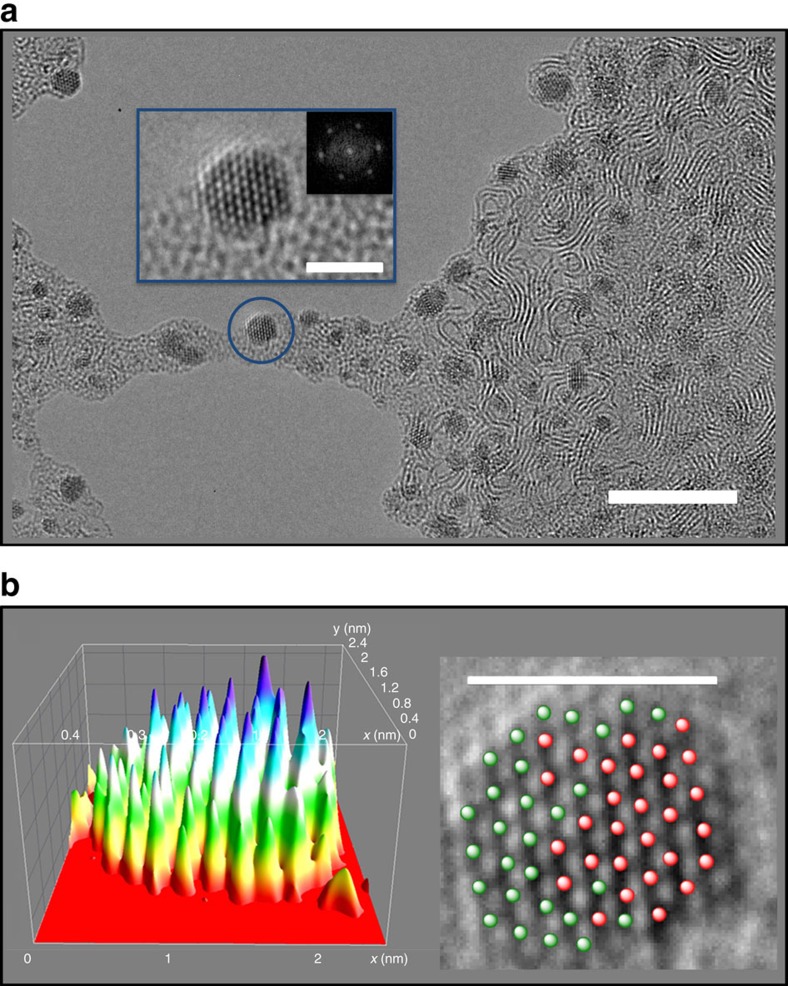
Ru-Os 3D-nanocrystals from RuOsMs micelles. (**a**) An array of mixed metal nanocrystals on the graphitic support formed after 60 min of irradiation (scale bar, 10 nm), with enlargement of the blue circled crystal showing atomic resolution (scale bar, 2 nm) and fast Fourier transform analysis of the hexagonal mixed metal crystal. The fast Fourier transform analysis of the hexagonal mixed metal crystal is also shown in the enlargement. (**b**) 3D projection (left) of the same crystal showing the difference of contrast between Ru, Os and the background (each peak corresponds to an atom, and the height/intensity of the peaks is dependent on the atomic TEM contrast; arbitrary colours), also depicted as 29 red (Os) and 28 green (Ru) balls on the 2D projection (right, scale bar, 2.1 nm). The presence of Ru and Os atoms was confirmed by a combination of scanning-TEM and energy-dispersive X-ray analysis.

To demonstrate the generality of this new technology, we encapsulated 1 mol equivalent of osmium complex **1** and 1 mol equivalent of its ruthenium analogue [Ru(*p*-cymene)(1,2-dicarba-*closo*-dodecarborane-1,2-dithiolate)] in **RuOsMs** micelles, spread them onto a lacey carbon grid and irradiated them with the electron beam in a similar procedure as described above. Again, it was possible to observe metal atom migration and nanocrystal formation on a timescale of *ca.* 1 h (Fig. 3).

The composition of the crystals was analyzed by a combination of scanning-TEM and energy-dispersive X-ray analysis at the single particle level (Supplementary Fig. 11); this clearly demonstrated their hetero-metallic Ru/Os nature. Interestingly, the Ru/Os molar ratios determined for 55 different crystals (0.91±0.07/1.13±0.07) are very close to the initial Ru/Os ratio in the **RuOsMs** micelles determined by inductively coupled plasma mass spectrometry (ICP-MS) (0.83±0.10/1.11±0.13). This suggests that the atomic percentage of each metal in the nanocrystals can be readily tuned by varying the ratio of metal complexes encapsulated in the micelles, opening-up new perspectives for the design of hetero-metal nanocrystals with defined size and composition.

## Discussion

We investigated the pattern and the rate of single Os movement by following the positions of a single Os atom over 200 s (20 frames with 10 s between each frame; Supplementary Fig. 12c, Supplementary Fig. 12d). The positions were extracted from the movie shown in Supplementary Video 4. Attempts were made to correlate the ‘hopping’ pattern with three different mechanisms: edge-to-edge, bridge-to-bridge and hole-to-hole hopping over the hexagonal graphitic matrix (Supplementary Fig. 12a,b). The closest fit corresponds to Os atoms moving from bridge-to-bridge (that is, sitting between a pair of two adjacent atoms) at a rate of 0.0089±0.0016, nm s^−1^. Such a bridge localization of Os atoms is apparent in some HR-TEM pictures (Supplementary Fig. 13), and is consistent with the favoured location of Pt atoms adsorbed on boron-doped graphene predicted by calculations^18^. Moreover, extrinsic point and line defects, such as foreign atoms at different positions, strongly modify the charge distribution and the electronic structure of graphene^32^. Density functional theory calculations have also suggested that graphitic boron dopants induce a deficiency of charge in graphene sheets, while sulfur doping of graphene is of particular interest as the resulting materials are expected to have a wider band gap than pure graphene^33^. Boron-doped graphene films show a large number of Stone-Wales defects^34^, while calculations indicate that sulfur doping induces a large local curvature that tends to increase the local reactivity^35^. Metal atoms have a high affinity for non-perfect and strained regions of point defects in graphene created by electron irradiation and annealing^36^. Thus, boron and sulfur centres and the resultant defects might act as centres, which attract Os atoms. This was confirmed by HR-TEM images showing that Os atoms are located in disturbed zones of the hexagonal pattern of the matrix (Supplementary Fig. 14). Individual Os atoms were also observed at step edges (Fig. 1d); similar observations have been reported for Au and Pt atoms on graphene layers^37^.

**Figure 4:**
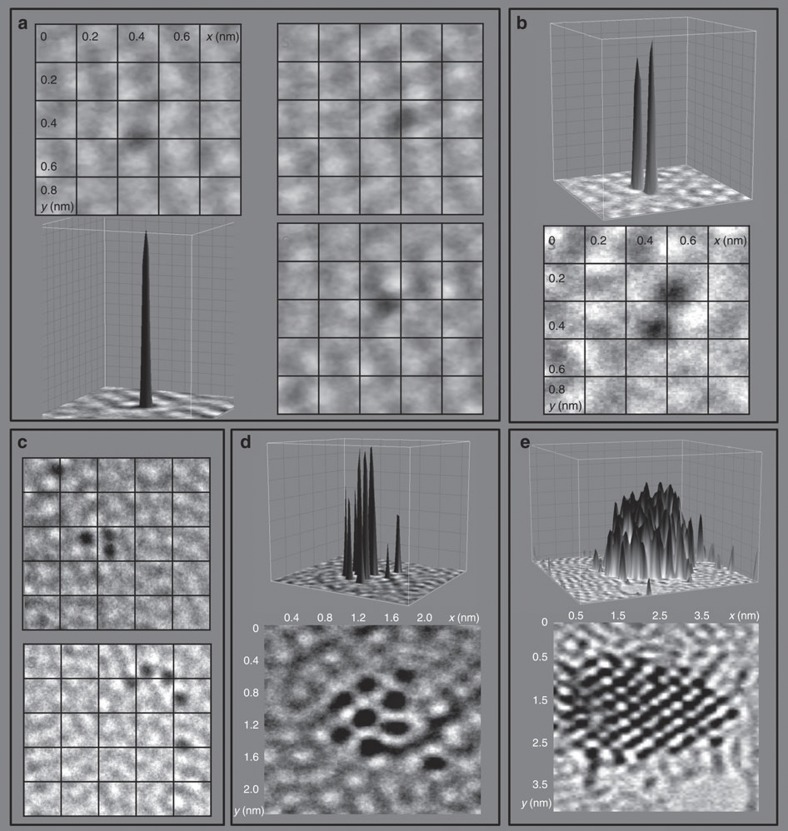
Atom-by-atom formation of osmium molecules clusters and eventually crystals. (**a**) 3D projection showing high contrast for a single Os atom, together with three 2D projections showing movement of the Os atom at a rate of 0.0177, nm s^−1^ over a period of 160 s. (**b**–**c**) Formation of dimer, trimer and tetramer molecules. (**d**) A cluster of eight Os atoms. (**e**) A ca. 25 Å crystal. All the images show the same region.

Such atom-by-atom fabrication of di- and poly-atomic molecules led to the rapid assembly of Os atoms into clusters. Figure 4 shows the atom-by-atom formation of osmium molecules, clusters made up of a few Os atoms and nanocrystals. This appears to be the first observation of atom-by-atom fabrication of nanocrystals.

In conclusion, our synthetic technology provides a new route to the *in situ* generation of an unsupported graphitic matrix together with metal atoms, which can migrate to form crystals. The method not only allows the multi-doping of the graphitic matrix, in this case with both boron and sulfur, but also captures the dynamics of metal cluster formation in real space with atomic precision. Indeed, this first report of the use of block copolymer micelles to encapsulate metal complexes for *in situ* reduction to metal atoms and formation of nanocrystals has allowed the dynamics of formation of metal nanocrystals to be observed, all the way from single atoms to molecules, clusters and then nanocrystals. This method has been exemplified by the production of a graphitic matrix doped with sulfur and boron, decorated with ångström-sized crystals of osmium, opening-up possible new perspectives for the design of nanoscopic highly-dense and pressure-resistant materials. The technology can readily be extended to expand the range of dopants in the supporting graphitic matrix, for example by replacing sulfur with selenium. It is also facile to fabricate a range of homo- and hetero-metal angstrom-size nanocrystals. We have illustrated this for mixed ruthenium–osmium crystals. Other combinations that might be readily accessible include Pd, Rh, Ir and Au, as the synthesis of the precursor carborane complexes of these metals is feasible. There is also wide scope for adapting this polymer-encapsulated metal complex synthetic procedure by variation of the block copolymer. The possibility of creating individual vacancies at desired locations in carbon nanotubes using electron beams has been recently demonstrated^38^, and might also be combined with our procedure to allow the grafting of Os nanocrystals onto specific hotspots. Our synthetic route, which gives rise to a self-supporting graphitic matrix, also offers attractive possibilities for studying the formation of multi-heteroatom-doped-graphitic sheets and the influence of dopants and defects without influence from the underlying support grids (for example, copper), a problem which often complicates the interpretation of metal deposition experiments. A combination of new experiments and computation will be necessary in future work for fully understanding the detailed mechanisms of doping, hopping, cluster rolling and coalescence. Finally, these nanocrystals may contribute to conceptual advances in the design of a new range of nanodevices, for example, for information storage, electronic circuitry, chemosensing and catalysis.

## Methods

### Materials

The preparation of the complex [Os(*p*-cym)(1,2-dicarba-*closo*-dodecaborane-1,2-dilthiolate)] (**1**) was based on a previous report^23^. The triblock copolymer P123 [poly(ethylene glycol)-*block*-poly(propylene glycol)-*block*-poly(ethylene glycol)] was purchased from Sigma-Aldrich and used as received. Anhydrous tetrahydrofuran (Aldrich) was used. Pure water (18.2 MΩ) was collected from a Purelab UHQ USF Elga system. Holey carbon grids with 200 mesh and lacey carbon grids were purchased from Quantifoil Micro Tools Gmbh and Elektron Technology UK Ltd, respectively and used as received.

### Synthesis of OsMs and OsRuMs

A tetrahydrofuran solution (1 ml) of complex **1** (5 mg ml^−1^) was added to an aqueous solution (10 ml) of polymer P123 (5 mg ml^−1^) and the resultant mixture was stirred at ambient temperature for 4 h. The solution was then dialyzed to remove the tetrahydrofuran (molecular weight cutoff=1000 Da), for 48 h, and then freeze-dried. A similar procedure was used for synthesizing **OsRuMs** with 1 mol equivalent of **1**, 1 mol equivalent of the Ru analogue and 1 mol equivalent of polymer P123.

### Characterization of the micelles OsMs and P123Ms

Dynamic light scattering (DLS) experiments (Supplementary Fig. 2) unambiguously demonstrated that polymer P123 and complex **1** self-assemble in aqueous solution. Encapsulation decreased the size of P123Ms micelles from 19.6±1.80 nm (hydrodynamic diameter, *D*_h_) to 11.5±2.35 nm for **OsMs** with a *Ð* of 0.03 (Supplementary Fig. 2a; Supplementary Table 1). Although micellar size usually increases after encapsulation of organic molecules^39^, incorporation of hydrophobic molecules can result in expulsion of water from the micelles, causing a contraction^40^. The hydrophobicity of **1** probably results in a stronger folding of the poly(ethylene oxide) chains around the complex through hydrophobic interactions, with concomitant expulsion of water from the core. A small second population of **OsMs** particles (<0.01% in number) was found at *D*_h_~\n220 nm, exhibiting a strong intensity in DLS analysis, owing to the aggregation of some particles (Supplementary Fig. 2c).

Cryogenic TEM (cryo-TEM) analysis without staining was then performed on Quantifoil carbon-coated grids to observe the morphology of the hydrophobic core (containing osmium complexes) of the nanoparticles in solution. The high contrast provided by the heavy osmium centres allowed facile imaging of the osmium-polypropylene glycol (PPG) core, but impaired the observation of the poly(ethylene glycol) corona owing to the polymer hydrated state; and disfavored by the small diameter of the micelles (Supplementary Fig. 3a), even after attempts to further stain the samples with uranyl acetate. From these experiments, it was clear that spherical micellar morphologies are formed when polymer P123 encapsulates complex **1**. The observed diameter of these **OsMs** nano-spheres is 7.85±1.97 nm (Supplementary Fig. 3b) with very low close-to-ideal dispersity, based on counting 157 particles counting (1.06, 1.00 being for ideal mono-disperse systems; see Supplementary Table 1). These data are in accordance with the *D*_h_ determined by DLS within experimental error.

To gain further insight into their structures in aqueous solution, and to confirm cryo-TEM and DLS results, **OsMs** and P123Ms were analyzed by synchrotron SAXS (Supplementary Fig. 4). The experimental profiles were fitted to three model functions for spherical micelles: SphereForm, CoreShellSphere, and PolyCoreShellRatio (PCR). The PCR model fitted excellently for both micelles with very low-*Ð* parameters (0.161 for **OsMs** and 0.146 for P123Ms, 0 being an ideal mono-disperse system, Supplementary Table 1). These analyses demonstrated that **OsMs** self-assembly leads to core/shell micelles with a core diameter of 9.06±0.12 nm, and a shell diameter of 6.50±0.15 nm (Supplementary Table 1). The core dimension of **OsMs** was larger than that of P123Ms (6.74±0.06 nm), while the corona dimension of **OsMs** was smaller than that of P123Ms shell (12.22±0.17 nm). The diameters of **OsMs** micelles by DLS and cryo-TEM are in accordance with the core diameter determined by SAXS within the experimental errors, while the diameters of P123 micelles from DLS and SAXS studies are similar.

From these data (scattering length density calculations, degrees of polymerization of Pluronic P123 and the molecular formulae of the polymer and of complex **1**; see Instrumentation and Methods), aggregation numbers for **OsMs** and P123Ms micelles were determined as 20±2 monomer chains per P123Ms micelle and 52±6 monomer chains per **OsMs** micelles. Determinations of osmium by ICP-MS gave a polymer/complex **1** ratio of 1/1±0.091 for **OsMs** showing that the 52 chains polymer chains self-assembled with 52±11 complexes **1** (see Supplementary Table 1). Similar core/shell diameters (Supplementary Table 1) with excellent fits to the PCR model were obtained from experiments at three different concentrations (1, 5, 10 mg ml^−1^; Supplementary Fig. 4a,b) for both **OsMs** and P123Ms. Hence concentration does not influence the micellar structure, a parameter of importance for Pluronic-type self-assemblies.

### Instrumentation

#### Ultraviolet-visible spectroscopy

Ultraviolet-visible absorption spectra were recorded on a temperature-controlled Varian CARY 300 Biospectrophotometer using 1-cm path-length quartz cuvettes (0.5 ml).

#### Inductively coupled plasma mass spectrometry

Osmium (^189^Os) content was determined using an ICP-MS Agilent technologies 7500 series instrument. The standard for osmium was purchased from Aldrich. Calibration curves were prepared using Os standard solutions in double deionised water with 3% nitric acid, ranging between 50 and 0.5 ppb (9 points). Samples were freshly prepared in double deionised water with 3% nitric acid. Readings were made in no-gas mode with a detection limit of 1 ppt for ^189^Os.

#### Dynamic light scattering

The *D*_h_ of nanoparticles was determined by DLS. Typically, an aqueous nanoparticle solution was measured with a Malvern Zetasizer NanoS instrument equipped with a 4 mW He-Ne 633 nm laser module at 25 °C. Measurements were carried out at a detector angle of 173° (back scattering). Data were analyzed by the Malvern DTS 6.20 software. *D*_h_ was calculated by fitting the apparent diffusion coefficient in the Stokes–Einstein equation *D*_h_=*kT*/(3*πηD*_app_), where *k* is the Boltzmann constant, *T* is the temperature and *η* is the viscosity of the solvent. *D*_h_ coincides with the hydrodynamic diameter when the sample is made of monodispersed spherical particles (*D*_app_ equals the translational diffusion *D*_t_).

#### Transmission electron microscopy

TEM was performed using a JEOL 2000FX operating at 200 kV. TEM samples were prepared by using holey and lacey carbon grids. One drop of the sample solution (5 μl) was applied to the grid and after 2 min the solution was blotted away before drying. Images were recorded on a Gatan Orius camera and were analyzed using ImageJ software. At least 100 particles from different parts of the grid were counted for each sample to obtain the average diameter.

#### Cryogenic-transmission electron microscopy

A JEOL 2010F TEM was operated at 200 keV and images were recorded on a Gatan UltraScan 4000 camera for cryo-TEM and glow discharge. The samples were prepared at ambient temperature by placing a droplet on a TEM grid. The extra liquid was then blotted with a filter paper and the grid was inserted in liquid ethane at its freezing point. The frozen samples were subsequently kept under liquid nitrogen.

#### High-resolution electron microscopy

A JEOL JEM-ARM200F HR-TEM was operated at 80 keV, 1.9 pA cm^−^2, with spherical aberration (*C*_s_) tuned to approximately +1 μm and images were recorded on a Gatan SC1000 Orius CCD camera.

#### Small-angle X-ray scattering

Measurements were carried out on the SAXS beamline at the Australian Synchrotron facility at a photon energy of 11 keV. The samples in solution were in 1.5 mm diameter quartz capillaries. The measurements were collected at a sample to detector distance of 3.252 m to give a *q* range of 0.004–0.2 Å^−1^, where *q* is the scattering vector and is related to the scattering angle (2*θ*) and the photon wavelength (*λ*) by the following equation (1):


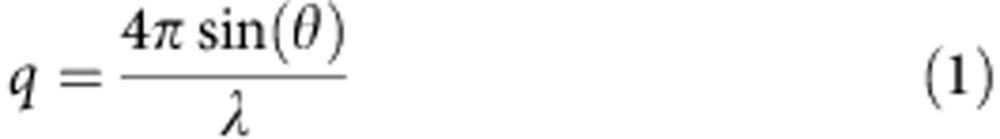


The scattering from a blank (H_2_O) was measured in the same location as sample collection and was subtracted for each measurement. All patterns were normalized to fixed transmitted flux using a quantitative beamstop detector. The scattering from a blank (THF/H_2_O) was measured in the same location as sample collection and was subtracted for each measurement. The two-dimensional SAXS images were converted in one-dimensional SAXS profiles (*I*(*q*) versus *q*) by circular averaging, where *I*(*q*) is the scattering intensity. Functions were used from the NCNR package, using Igor software. Scattering length densities were calculated using the ‘Scattering Length Density Calculator’ provided by NIST Centre for Neutron Research.

## Author contributions

N.P.E.B. and P.J.S. conceived and obtained funding for the project, designed the experiments, oversaw the research and wrote the paper. R.B. and A.P.B. designed and performed the experiments, oversaw the research and wrote the paper. R.K.O.R. suggested key experiments, and discussed the results. A.P.D., R.J.P., J.J.S.B., A.M.S., N.K., I.H.P. and C.J.S. performed the experiments. All authors discussed the results and commented on the manuscript.

## Additional information

**How to cite this article:** Barry, N. P. E. *et al.* Fabrication of crystals from single metal atoms. *Nat. Commun.* 5:3851 doi: 10.1038/ncomms4851 (2014).

## Supplementary Material

Supplementary Figures and Table Supplementary Figures 1-14 and Supplementary Table 1

Supplementary Movie 1 Cluster rolling. The rolling of an Os cluster on a multi-doped graphitic surface

Supplementary Movie 2 Cluster fusions. Motion, merging and fusion of Os clusters

Supplementary Movie 3 3D projection. The 3D projection of an Os clusters on a multi-doped graphitic surface

Supplementary Movie 4 Atom Hopping. The hopping of an individual Os atom on a multi-doped graphitic surface
