# Supplementary material for: Fabrication of crystals from single metal atoms
Source: Nat Commun. 2014 May 27;5:3851. doi: 10.1038/ncomms4851 (PMC4050283; doi:10.1038/ncomms4851)
Supplement: Supplementary Figures and Table — Supplementary Figures 1-14 and Supplementary Table 1 [file ncomms4851-s1.pdf]

SUPPLEMENTARY INFORMATION

Supplementary Figures

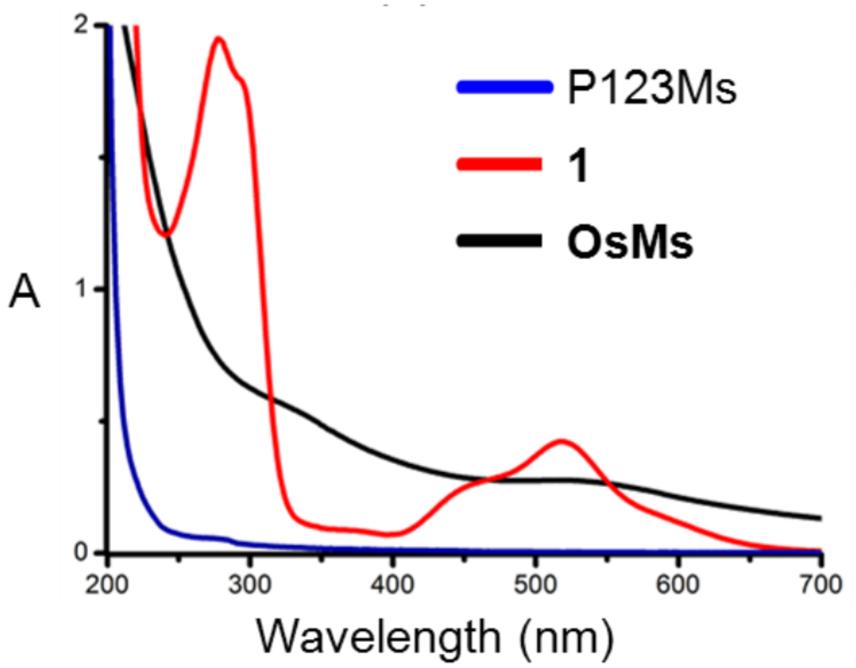

Supplementary Figure 1. UV-visible spectra: P123Ms (10^{-4} M in H_2O; blue line), complex **1** (10^{-3} M in CH_3CN; red line), and **OsMs** (10^{-4} M in H_2O; black line). The change of color from transparent P123Ms to purple **OsMs** shows that **1** is encapsulated in the polymer micelles.

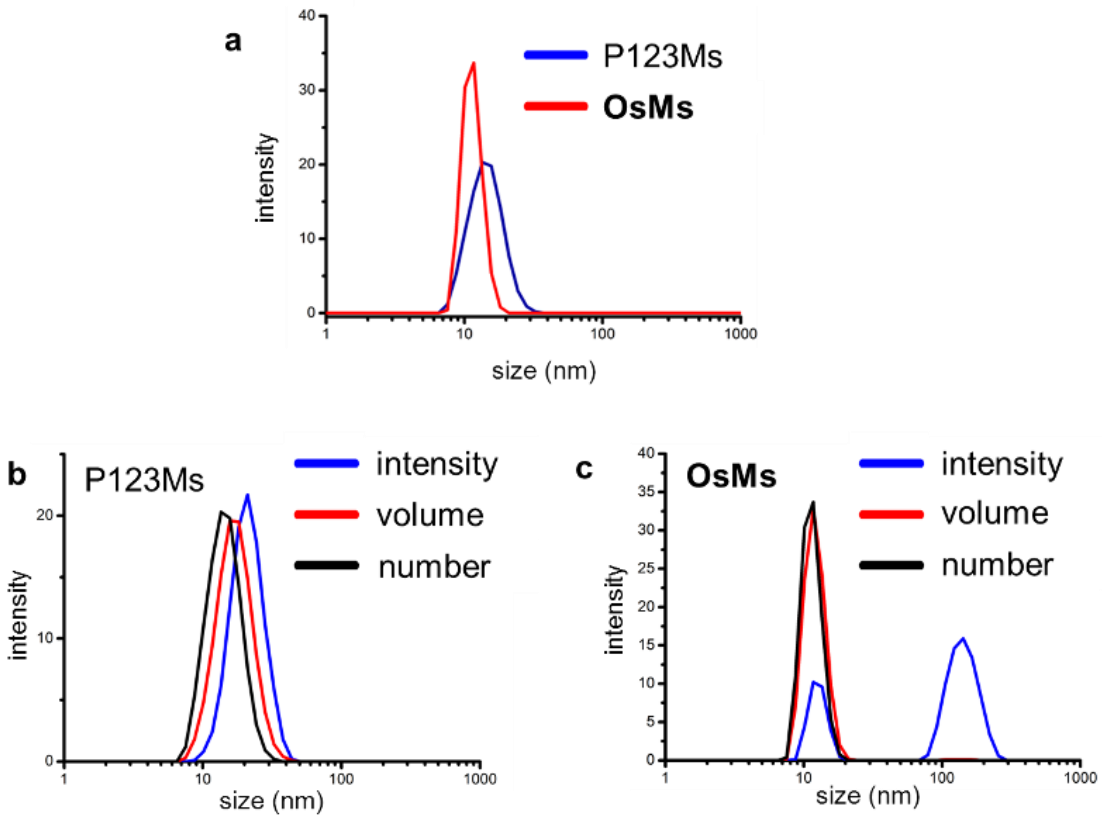

Supplementary Figure 2. Characterization of polymer micelles by dynamic light scattering (DLS). (**a**) Size distribution in number of P123Ms and **OsMs** (10 mg/mL in H_2O); Intensity, volume and number distribution for (**b**) P123Ms, and (**c**) **OsMs**; 10 mg/mL, H_2O. These experiments show that polymer P123 and complex **1** self-assemble in aqueous solution.

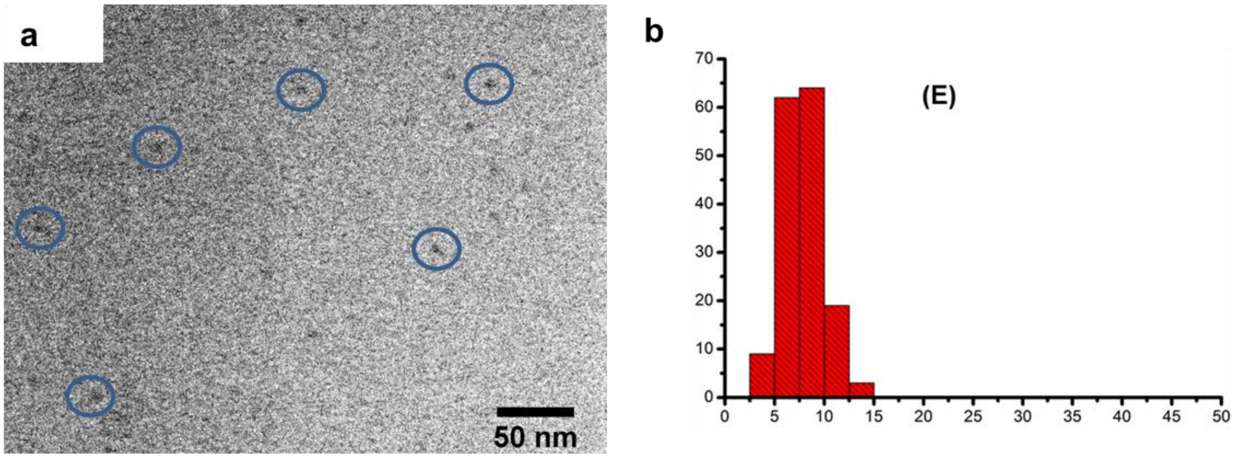

Supplementary Figure 3. Characterization of polymer micelles by cryogenic-TEM (cryo-TEM). (**a**) Cryo-TEM image of OsMs. (**b**) Particle counting/histogram analysis of OsMs from cryo-TEM images.

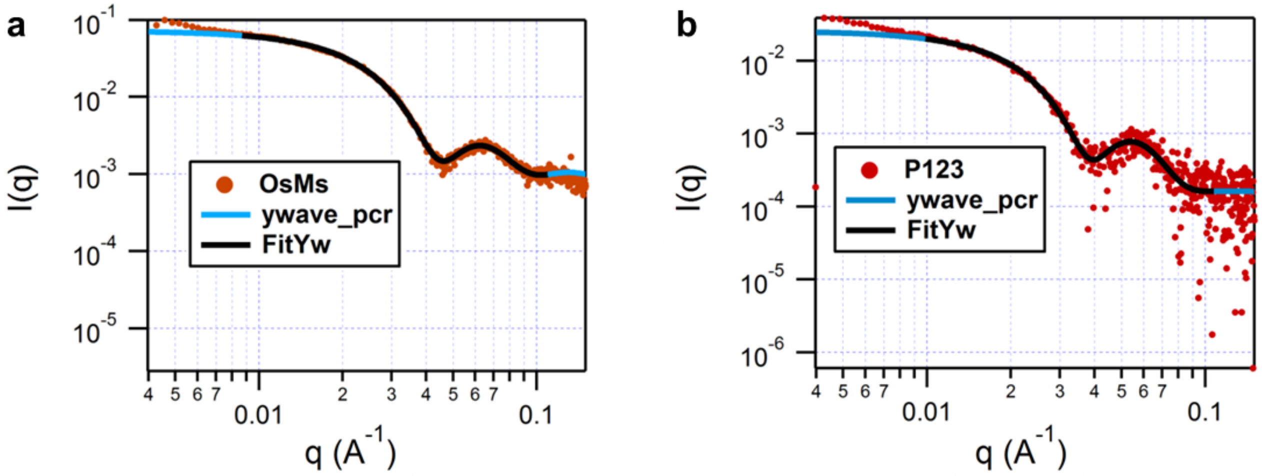

Supplementary Figure 4. Characterization of polymer micelles by small-angle x-ray scattering (SAXS). (**a**) Small-angle X-ray scattering (SAXS) experimental profiles and fitting with the PolyCoreShellRatio model of micelles OsMs and (**b**) P123Ms: 1 mg/mL aqueous solutions.

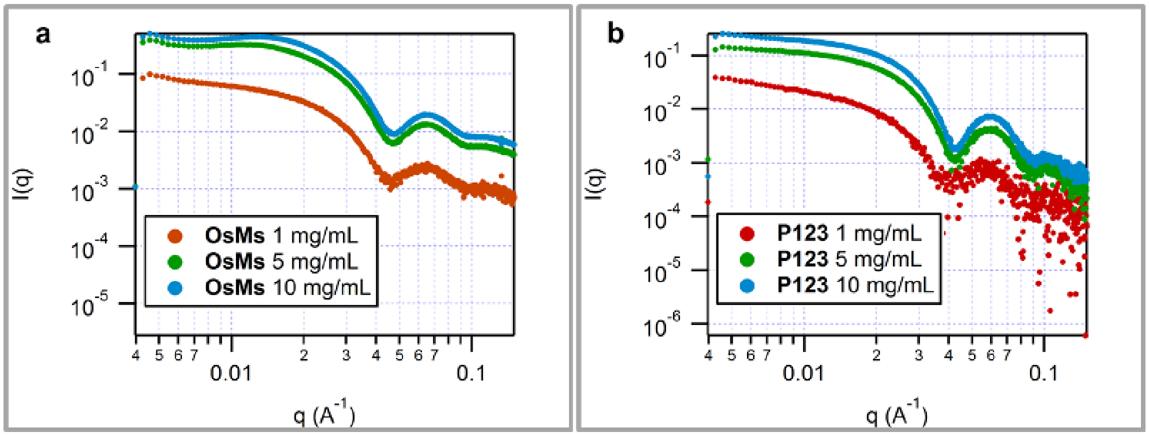

Supplementary Figure 5. Small-angle X-ray scattering (SAXS) profiles. (**a**) **OsMs** and (**b**) P123Ms, at three different concentrations (1; 5; 10 mg/mL) showing small structure-factor effects at higher concentrations but the micellar structure which does not change significantly with concentration for both P123Ms and **OsMs**.

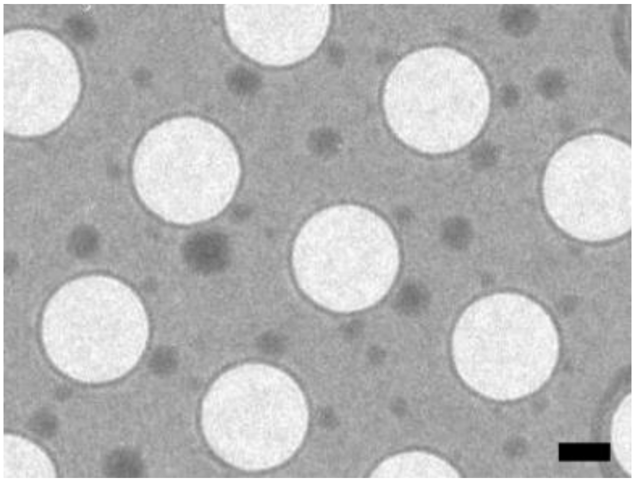

Supplementary Figure 6. Dry-state TEM images of the self-assembled polymer OsMs micelles on Quantifoil® grids (scale bar: 1 µm), demonstrating that **OsMs** spread and form stable nanostructures in the dry-state (with a diameter *ca.* 30 fold larger in the dry-state than for spherical micelles in solution)

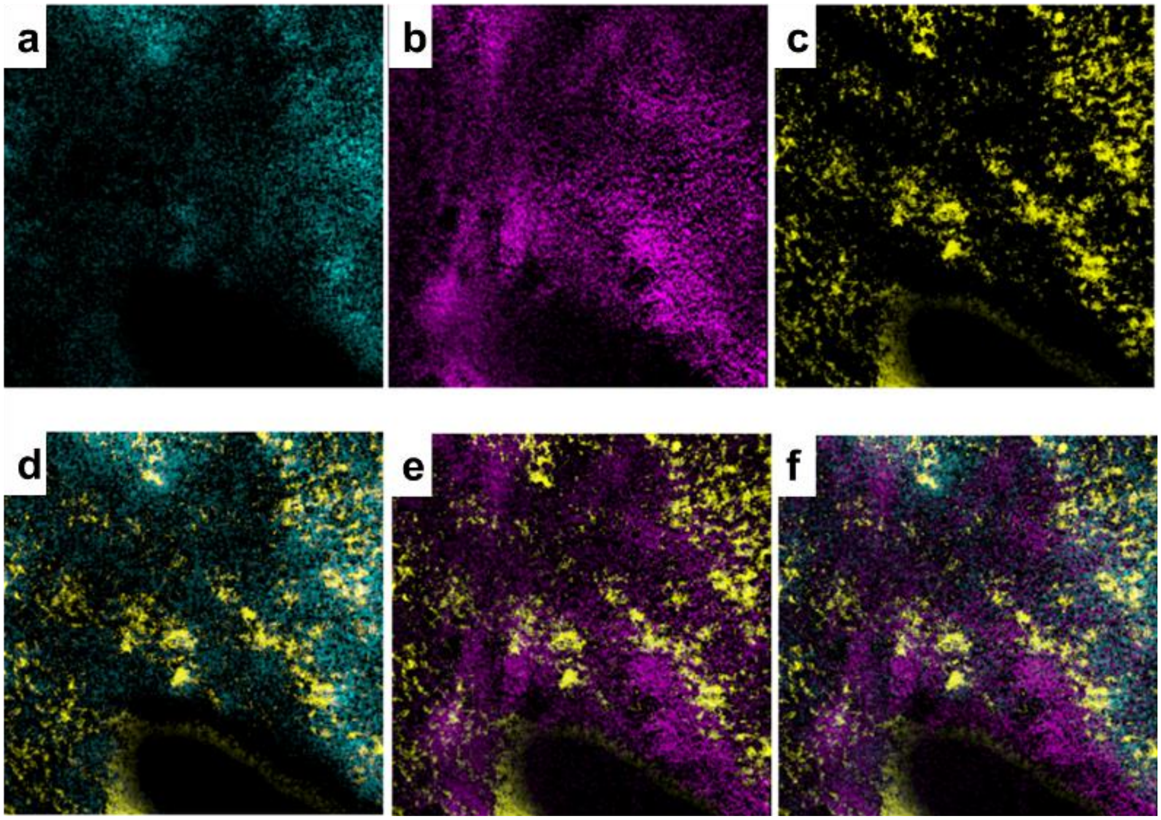

Supplementary Figure 7. Electron energy loss elemental mapping of nanoclusters and self-supported graphitic matrix. (**a**) Sulfur L, (**b**) boron K, and (**c**) osmium O maps. (**d**) Osmium and sulfur superimposed maps. (**e**) Osmium and boron superimposed maps. (**f**) Osmium, sulfur, and boron superimposed maps. On each map, the element(s) appear(s) as bright spots when present. For instance, osmium atoms are localized in regions of crystals, and appear to be close to high-boron and high-sulfur sites, although boron and sulfur atoms are also observable throughout the lattice. All the images show the same region.

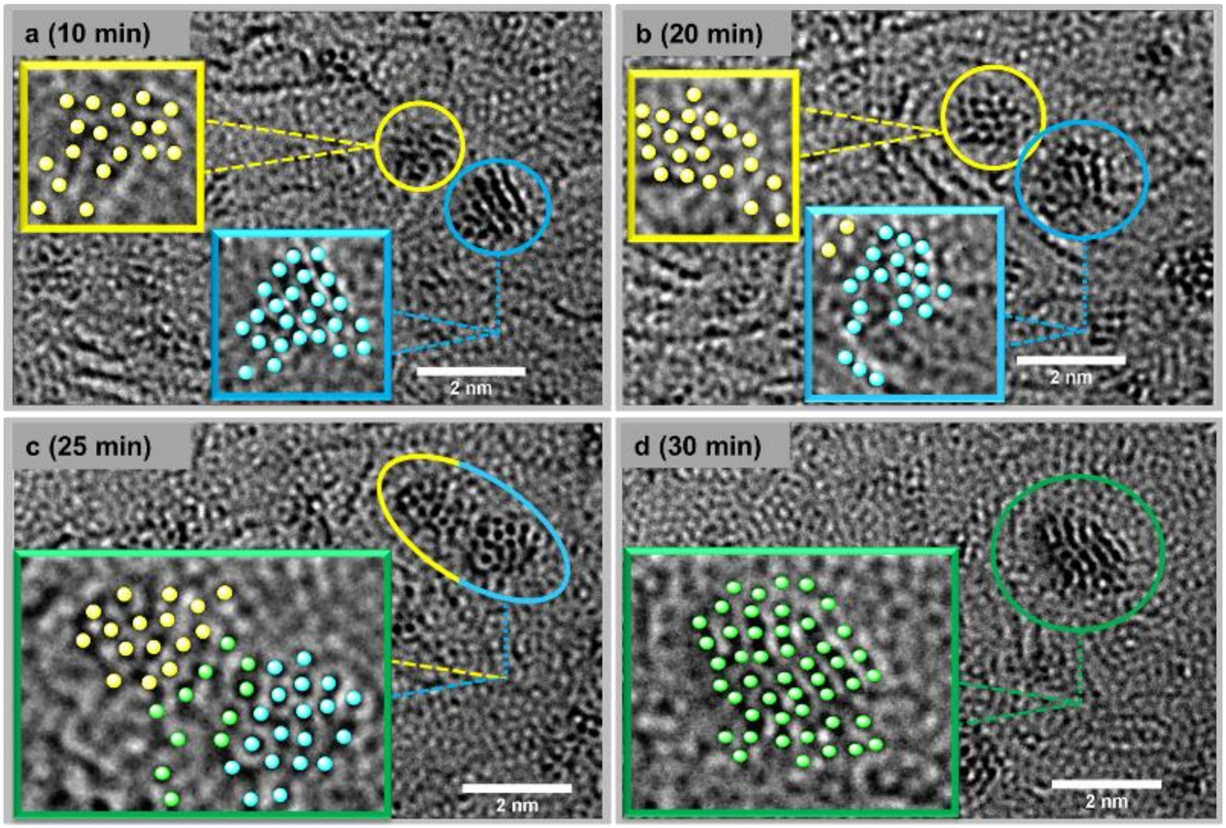

Supplementary Figure 8. Migration and fusion of energized osmium clusters leading to the growth of nanocrystals. Images recorded after electron beam irradiation times of (**a**) 10 min, (**b**) 20 min, (**c**) 25 min, and (**d**) 30 min, showing the fusion of two clusters (yellow and blue balls for Os atoms of each cluster) into a larger fused cluster (green balls).

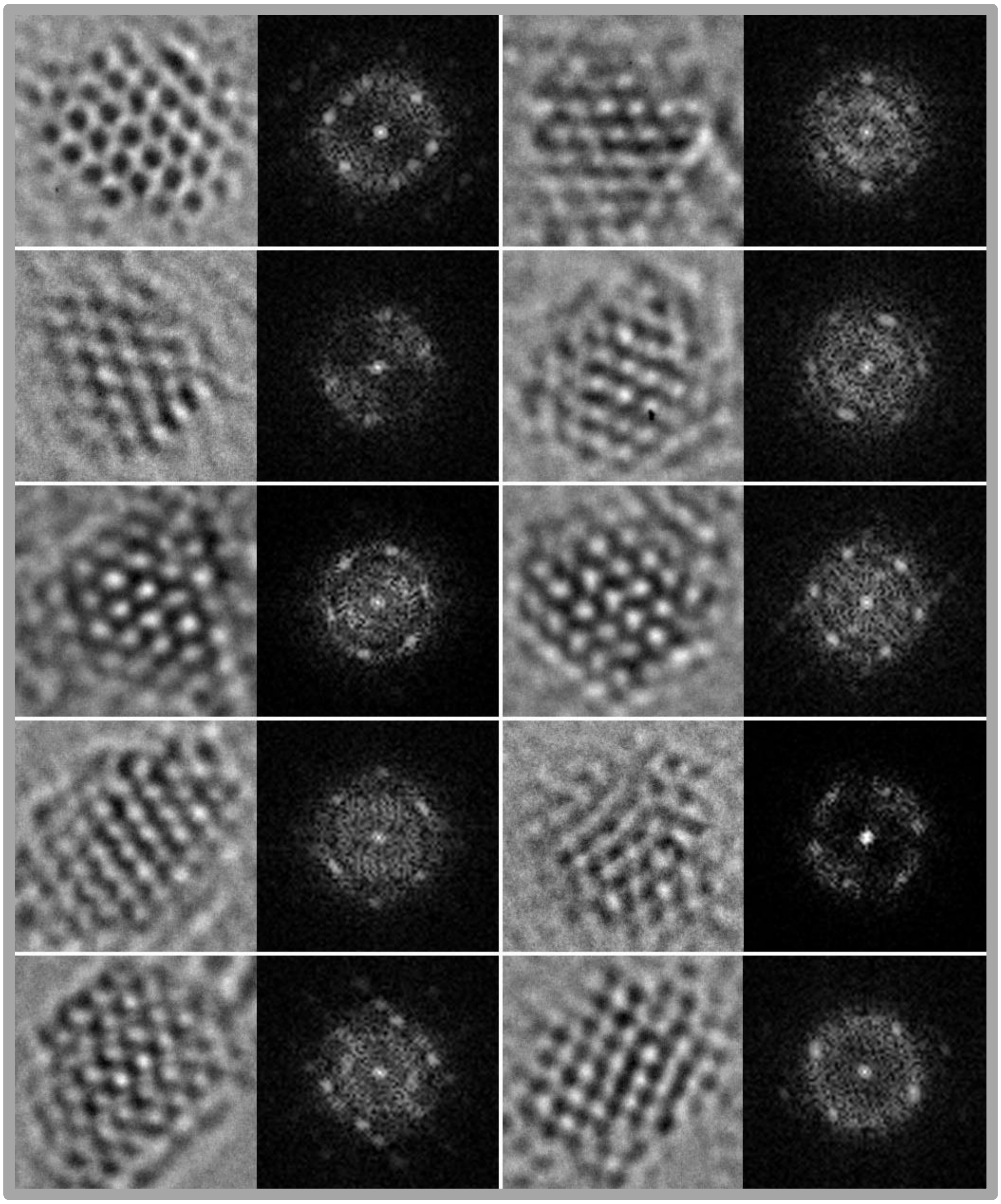

Supplementary Figure 9. Montage of 10 Os nanocrystals with their FFT analysis.

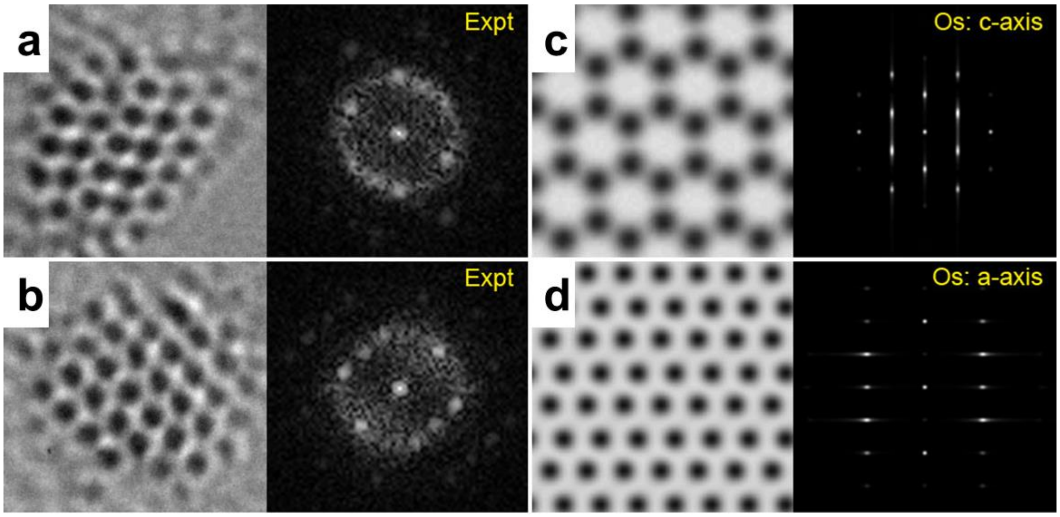

Supplementary Figure 10. Experimental and simulated crystal structure comparisons. (**a.** and **b**) Two nanocrystals with close resemblance to hexagonal structure. (**c.** and **d**) Simulations along the c-axis and a-axis, respectively.

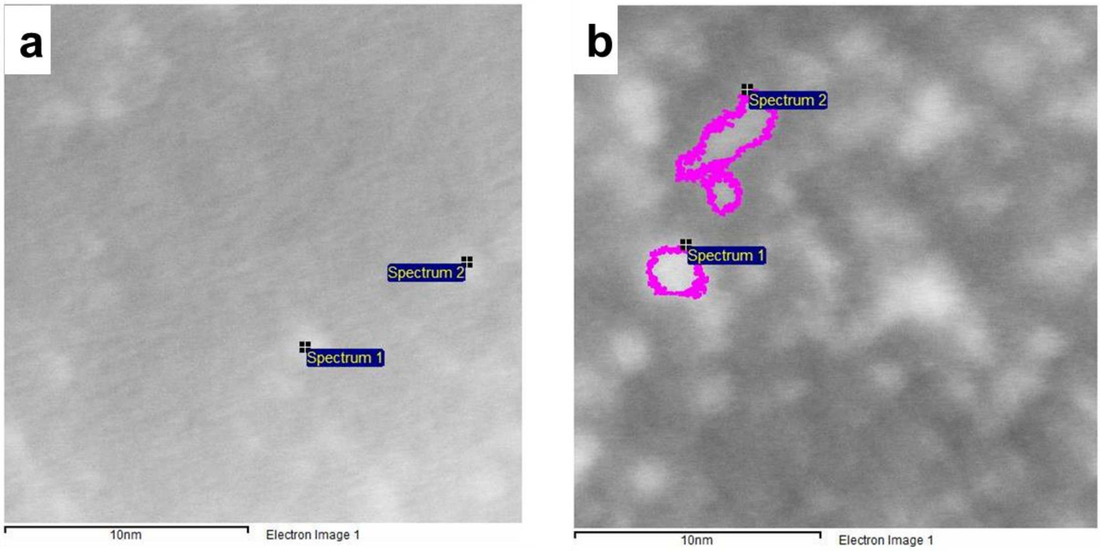

Spectrum	C (weight%)	O (weight%)	S (weight%)	Cu (weight%)	Ru (weight%)	Os (weight%)	Ru/Os (molar ratio)
**(a)-1**	49.22	5.72	1.11	30.23	5.00	8.30	**1.13**
**(a)-2**	44.20	9.46	1.08	34.19	3.87	7.21	**1.01**
**(b)-1**	32.41	10.51	0.70	38.39	5.98	12.00	**0.94**
**(b)-2**	45.58	10.95	1.10	34.42	2.53	5.42	**0.88**

Supplementary Figure 11. Combination of scanning-TEM (STEM) and energy-dispersive X-ray (EDX) analysis. (**a.** and **b**) Two areas are shown; the bright spots correspond to nanocrystals. The table shows the EDX analysis for four crystals, along with the Ru/Os molar ratio.

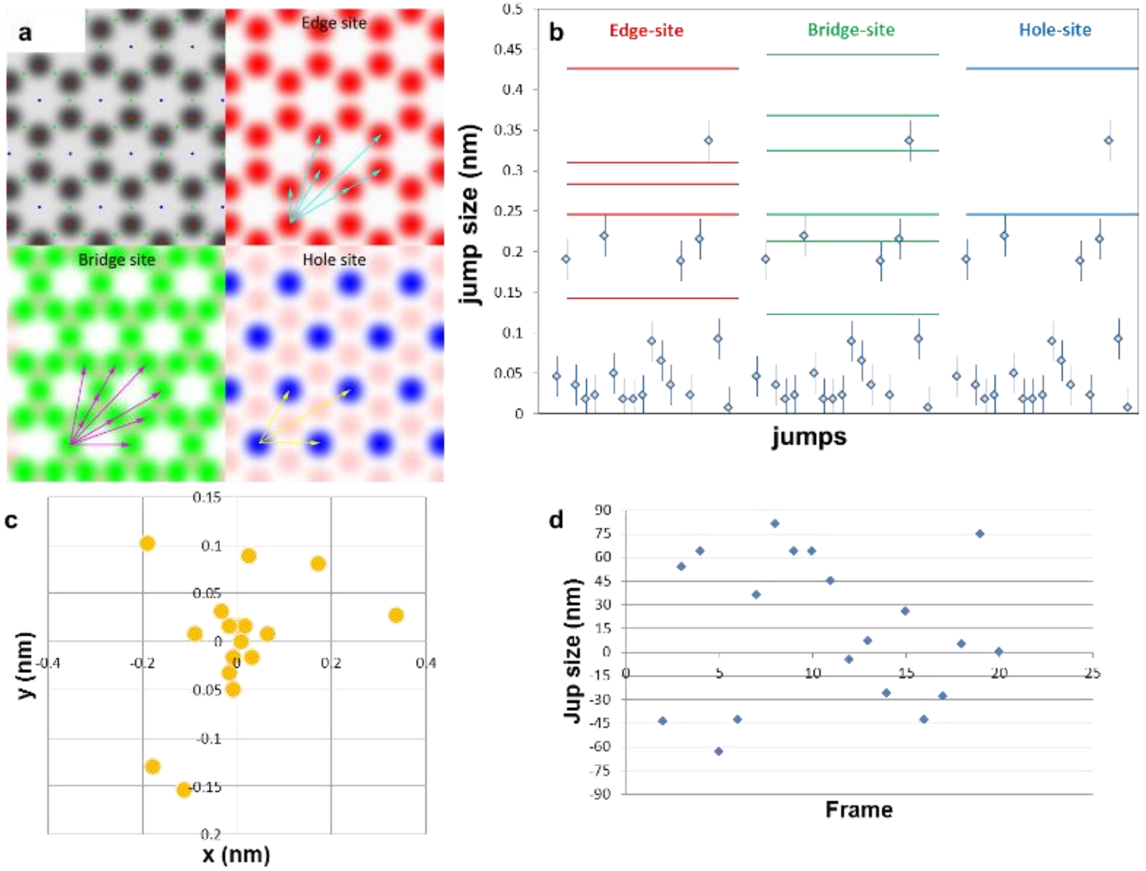

Supplementary Figure 12. Pattern of single Os atom 'hopping'. (**a**) A hexagonal graphene lattice and the three possible atomic positions (edge = red; bridge = green; hole = blue). Each type has a distinct set of vectors between equivalent sites, indicated by arrows. (**b**) Each dot represents the observed jump size for one single Os atom in a time frame of 200 seconds (each point represents one jump, and 20 jumps were considered); Horizontal lines correspond to the theoretical jump size (*i.e.* vector lengths in **a**) for edge-to-edge (red lines), bridge-to-bridge (green lines), and hole-to-hole (blue lines) patterns of hopping. (**c**) Changes of position and (**d**) jump direction for this single Os atom in the same time frame of 200 s (20 frames, with 10 seconds between each frame).

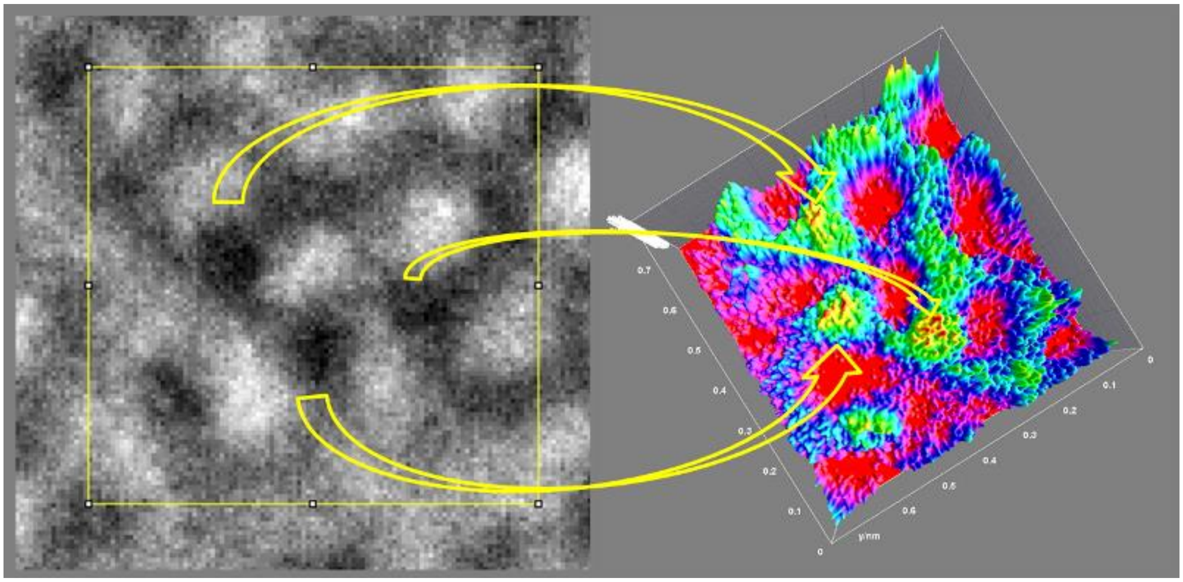

Supplementary Figure 13. HR-TEM image of a cluster of 3 Os atoms, and 3D projection. In the projection, the Os atoms can be seen because of their higher contrast as compared to the graphitic matrix (yellow-red peaks).

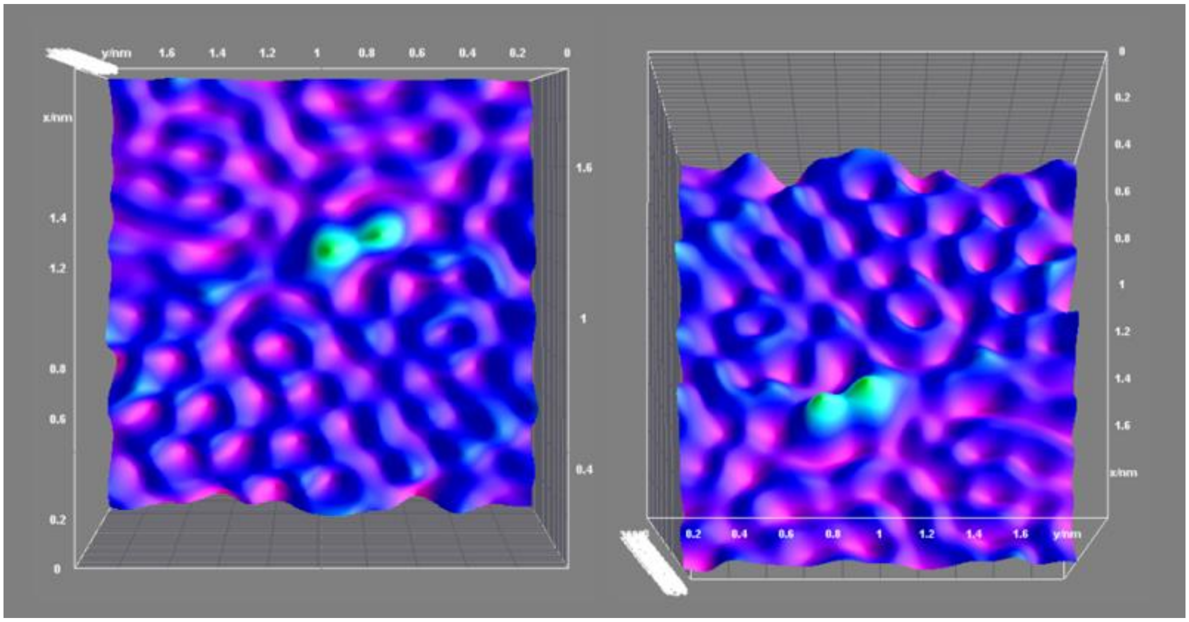

Supplementary Figure 14. Two views of a dinuclear Os_2 molecule on the distorted graphitic matrix. The two Os atoms are in light blue.

Supplementary Table

**Micelles**	**OsMs**	**P123Ms**
Polymer aggregation number	52±6	20±2
Os complexes per micelle	52±11	0
DLS diameter (nm)	11.50±2.35	19.60±1.80
DLS dispersity	0.030	Not determined
Cryo-TEM diameter (nm)	7.85±1.97	Not determined
Cryo-TEM dispersity	1.06	Not determined
SAXS total diameter (nm)	15.56±0.27	18.96±0.23
SAXS core diameter (nm)	9.06±0.12	6.74±0.06
SAXS shell diameter (nm)	6.50±0.15	12.22±0.17
SAXS dispersity	0.161	0.146

Supplementary Table 1. Physical characteristics of OsMs and P123Ms micelles. The parameters were obtained from DLS, cryo-TEM and SAXS data at 1 mg micelles/mL.
